# Draft Genome Sequences of Pseudomonas MWU13-2625 and MWU12-2115, Isolated from a Wild Cranberry Bog at the Cape Cod National Seashore

**DOI:** 10.1128/MRA.00992-18

**Published:** 2018-09-27

**Authors:** Ghazal Ebadzadsahrai, Jonathon Thomson, Scott Soby

**Affiliations:** a Biomedical Sciences Program, College of Graduate Studies, Midwestern University, Glendale, Arizona, USA; b College of Dental Medicine, Midwestern University, Glendale, Arizona, USA; c Biomedical Sciences Program, College of Graduate Studies and College of Veterinary Medicine, Midwestern University, Glendale, Arizona, USA; Georgia Institute of Technology

## Abstract

Two highly similar Pseudomonas sp. genome sequences from wetland bog soil isolates with draft genomes of ~6.3 Mbp are reported. Although the exact taxonomic placement and environmental roles of these bacteria are unclear, predicted genes for stress tolerance, antibiotic resistance, and a type VI secretion system were detected.

## Abstract (precis)

Two highly similar Pseudomonas sp. genome sequences from wetland bog soil isolates with draft genomes of ~6.3 Mbp are reported.

## ANNOUNCEMENT

**FIG 1:**
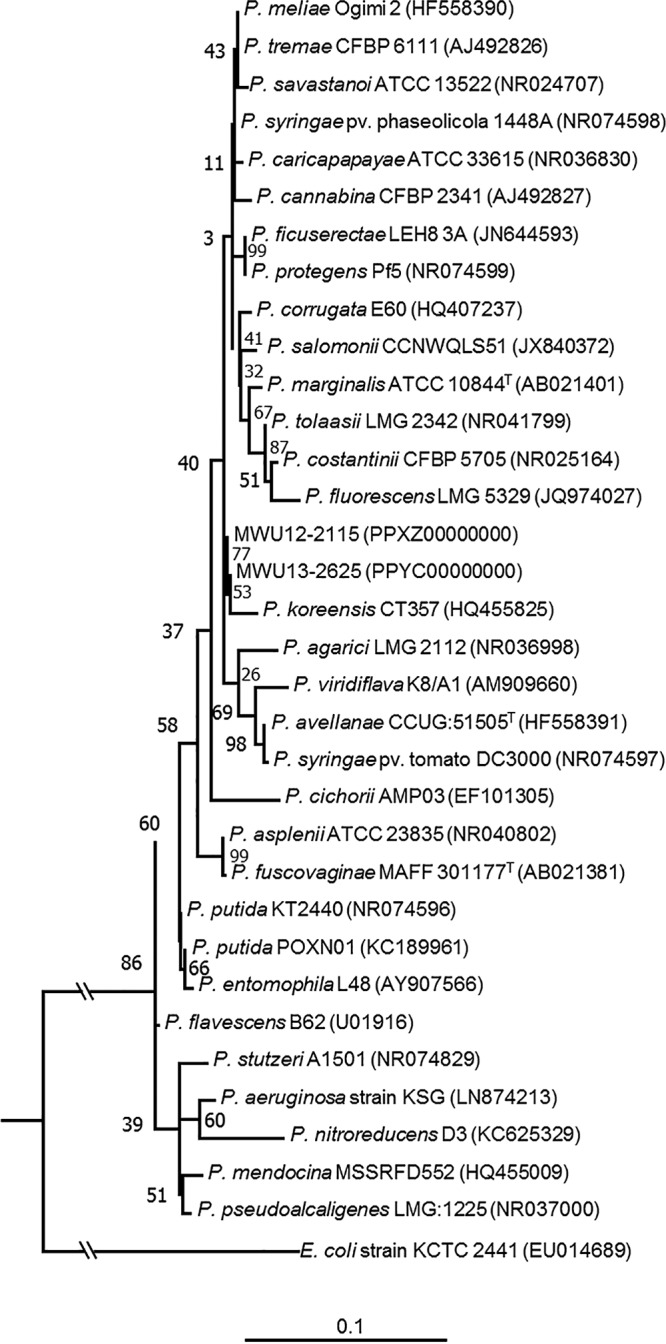
16S rRNA molecular phylogenetic analysis of representatives of the genus Pseudomonas. The evolutionary history of the genus Pseudomonas was inferred with the maximum likelihood method based on the Kimura two-parameter model with elimination of all positions containing gaps. The tree based on a total of 1,070 positions within the 16S rRNA gene with the highest log likelihood (−2,995.61) is shown. The percentage of trees in which the associated taxa clustered together is shown at each node. The initial tree or trees were generated with neighbor-join and BIONJ algorithms to estimated pairwise distances using maximum composite likelihood and then selecting the topology with the highest log likelihood value. Evolutionary rate differences between sites were modeled using a discrete gamma distribution (+G, parameter = 0.5039), allowing for some sites to be invariable ([+I], 82.26% sites). The tree is drawn to scale, with branch lengths measured in the number of substitutions per site, except for the Escherichia coli outgroup branch, which contains 12 total substitutions from the divergent branch point. The analysis involved 34 nucleotide sequences.

Microbes play an important role in wetland ecology, but little is known about the bacteria or how they interact with these ecosystems. A culture-dependent survey of bacteria in bog soils at the Cape Cod National Seashore in Massachusetts yielded a large number of Pseudomonas sp. isolates of uncertain taxonomic placement. Two of these isolates, MWU13-2625 and MWU12-2115, are 83.7% similar by digital DNA-DNA hybridization (dDDH) (1). They clustered with Pseudomonas koreensis with 16S rRNA phylogeny (Fig. 1) (2), but with genomic analysis using dDDH (44%) (1, 3) and the Orthologous Average Nucleotide Identity (OrthoANI) tool (91%) (4, 5), they fall outside the P. koreensis taxon.

Bacteria were seeded on King’s medium B (KMB) agar containing 50 mg ml^−1^ cycloheximide and ampicillin and grown at 26°C, followed by 3× single-colony purification on KMB. Genomic DNA (gDNA) was extracted from overnight KMB broth cultures using a DNeasy blood and tissue kit (Qiagen) and sequenced at the Arizona State University CLAS Genomics Core facility. gDNA was sheared to approximately 600-bp fragments (Covaris M220 focused-ultrasonicator; Thermo Fisher), and libraries were constructed using a Kapa Biosystems preparation kit. DNA fragments were end repaired and A tailed (Kapa protocol). Combination indexes/adapters (Bioo Scientific) were ligated onto each sample and multiplexed into a single lane. AMPure beads (Agencourt Bioscience/Beckman Coulter) were used to clean adapter-ligated molecules, which were then amplified with a Kapa HiFi enzyme. Libraries were quantified with quantitative PCR (qPCR) (Kapa kit KK4835) and analyzed on an Agilent Bioanalyzer before multiplex pooling. Libraries were sequenced in 2 × 300- and 2 × 150-bp paired-end (PE) flow cells on the MiSeq platform (Illumina).

Whole-genome sequences were processed using the Comprehensive Genome Analysis pipeline available on the PATRIC website (https://www.patricbrc.org) (6) using default parameters. The genome of MWU13-2625 had a sequence coverage of 132× and was assembled into 56 contigs with a total length of 6,270,237 bp (*N*_50_, 465,430 bp; G+C content, 60.4%). The genome of MWU12-2115 had a sequence coverage of 151× and contains 51 contigs with a total length of 6,351,070 bp (*N*_50_, 395,905 bp; G+C content, 60.3%).

Genome annotation was performed using the RASTtk function of PATRIC (4). Isolate MWU13-2625 contains 5,803 coding DNA sequences (CDSs) with 74 tRNA and 7 rRNA operons. Pseudomonas MWU12-2115 contains 5,955 CDSs with 69 tRNA and 5 rRNA operons. The genome is predicted to encode antibiotic resistance genes for fosfomycin resistance, β-lactamases, and the multi-antimicrobial extrusion protein (MATE) family of multidrug resistance efflux pumps. There are 69 and 71 iron uptake genes, 13 and 15 toxin-antitoxin systems, and 4 and 5 motility genes in MWU13-2625 and MWU12-2115, respectively. There were eight endolysin (murein hydrolase) genes and *rhs* genes for type VI secretion clusters with potential secreted proteins Hcp and VgrG, two hallmark proteins of the type VI secretion system (T6SS) (7) in each isolate. No prophages were detected in either isolate with PHAST (http://phast.wishartlab.com) (8).

### Data availability.

The whole-genome sequences have been deposited at DDBJ/EMBL/GenBank under the accession number PPYC00000000 for Pseudomonas MWU13-2625 and PPXZ00000000 for Pseudomonas MWU12-2115. The versions described in this paper are versions PPYC02000000 and PPXZ02000000, respectively. The SRA accession numbers are SRX4480119 and SRX4452408.
